# An immunogenomic stratification of colorectal cancer: Implications for development of targeted immunotherapy

**DOI:** 10.4161/2162402X.2014.976052

**Published:** 2015-04-02

**Authors:** Neeraj Lal, Andrew D Beggs, Benjamin E Willcox, Gary W Middleton

**Affiliations:** 1 Cancer Immunology and Immunotherapy Centre; School of Cancer Sciences; University of Birmingham; Birmingham, UK

**Keywords:** colorectal cancer, immune signature, microsatellite instability, *RAS*, stratification, CIRC, Co-ordinate Immune Response Cluster, CMS, consensus molecular subtypes, CRC, colorectal cancer, CRCSC, colorectal cancer subtyping consortium, MSI-H, microsatellite unstable (high), MSI-L, microsatellite unstable (low), MSS, microsatellite stable, TCGA, The Cancer Genome Atlas, TILs, tumor-infiltrating lymphocytes

## Abstract

Although tumor infiltrating lymphocyte (TIL) density is prognostic and predictive in colorectal cancer (CRC), the impact of tumor genetics upon colorectal immunobiology is unclear. Identification of genetic factors that influence the tumor immunophenotype is essential to improve the effectiveness of stratified immunotherapy approaches. We carried out a bioinformatics analysis of CRC data in The Cancer Genome Atlas (TCGA) involving two-dimensional hierarchical clustering to define an immune signature that we used to characterize the immune response across key patient groups. An immune signature termed The Co-ordinate Immune Response Cluster (CIRC) comprising 28 genes was coordinately regulated across the patient population. Four patient groups were delineated on the basis of cluster expression. Group A, which was heavily enriched for patients with microsatellite instability (MSI-H) and POL mutations, exhibited high CIRC expression, including the presence of several inhibitory molecules: *CTLA4*, PDL1, PDL2, *LAG3*, and *TIM3*. In contrast, *RAS* mutation was enriched in patient groups with lower CIRC expression. This work links the genetics and immunobiology of colorectal tumorigenesis, with implications for the development of stratified immunotherapeutic approaches. Microsatellite instability and POL mutations are linked with high mutational burden and high immune infiltration, but the coordinate expression of inhibitory pathways observed suggests combination checkpoint blockade therapy may be required to improve efficacy. In contrast, *RAS* mutant tumors predict for a relatively poor immune infiltration and low inhibitory molecule expression. In this setting, checkpoint blockade may be less efficacious, highlighting a requirement for novel strategies in this patient group.

## Introduction

The density of TILs and the expression of certain immune-related genes are of prognostic and predictive value in CRC.^1–5^ However, the factors that determine a patient's immune phenotype are unclear, and few systematic analyses have investigated the somatic and germline molecular drivers of immune infiltration. Although, microsatellite unstable (MSI-H) cancers are known to be associated with increased TIL density,^6-12^ the nature of the immune infiltration and the molecular drivers of the immune phenotype in microsatellite stable (MSS) CRC are poorly understood. In particular, it is unclear whether defined molecular subsets (*RAS* mutant, *BRAF* mutant, *PIK3CA* mutant, quadruple wildtype (*BRAF*, *PIK3CA*, *NRAS*, *KRAS* all wildtype)) are associated with high or low immune infiltration. In addition, in both MSI-H and MSS cancers, the extent to which therapeutically tractable inhibitory immune checkpoint receptors are represented is unclear and is of substantial interest, particularly considering recent checkpoint blockade failures in CRC.^13,14^ Given the prognostic and predictive relevance of CRC immunophenotype, a clearer understanding of the link between immunophenotype with tumor genotype is crucial.

In this study, we carried out a bioinformatics analysis of CRC data in The Cancer Genome Project (TCGA). Our analyses defined a co-regulated cluster of immune related genes with a distinct *Th1* bias**,** expression of which defines four patient subgroups. The findings have implications for our understanding of the immunobiology of colorectal carcinogenesis, and for development of stratified immunotherapy approaches for CRC.

## Results

### Coordinate expression of immune response-related genes in colorectal cancer

We interrogated expression of immune response-related genes in the CRC dataset in the TCGA (*n* = 195). Preliminary analyses focused on an initial gene list (**Table S1**) based partly on previous studies on bio-molecular networks incorporating immune genes linked with disease-free survival in CRC.^1,4^^,^^5^ These included those associated with *Th1* subset function (*STAT1*, *IRF1*, *IFNG*, *TBX21*, *IL18RAP*, *ICOS*, *GNLY*), certain chemokines (CX3CL1, *CXCL9*, *CXCL10*), adhesion molecules (ICAM and MADCAM), and an array of class II genes. Also, we included a number of immune checkpoint genes (PD-1, PD-L1, PD-L2, LAG3, TIM3, CTLA-4), two of which (PD-L1 and PD-L2) were previously associated with outcome.^4^ Finally**,** our initial gene list was supplemented with class I genes and additional class II genes and genes involved in T cell activation, together with NKG2D ligands and related genes relevant to innate immune activation (e.g. ULBPs, *PROCR*^15^).

We performed unsupervised two-dimensional hierarchical clustering to assess the extent to which gene expression was co-ordinate or independent across the patient cohort. Visual analysis of the clustering highlighted a 28-gene subset (**Table 1**) which formed a clear gene grouping, expression of which was coordinately regulated (**Fig. S1**). Gene-tree analysis of the dendrogram confirmed the validity of this grouping, identifying a subset of 24 highly coordinated genes (distance threshold 0.46) predominantly associated with *Th1* immunity, including numerous class II MHC loci, and inhibitory molecules targeted in checkpoint blockade strategies. This 24-gene block was identical to our 28-gene cluster other than the absence of three additional class II MHC loci (*HLA-DRB5*, *HLA-DQA2*, and *HLA-DQA1*) and one additional inhibitory molecule (*HAVCR2* (TIM3)), all of which correlated closely with the 24-gene block and were positioned directly adjacent on the gene cluster dendrogram. We therefore, proceeded with the 28-gene cluster as it formed a clearly coordinated block on visual and correlation analysis. We termed this grouping the CIRC.

**Table 1.** Genes within the coordinate immune response cluster (CIRC). These genes are presented in the order of the CIRC signature

Gene ID
*HLA-DQA1*
*HLA-DQA2*
*HLA-DRB5*
*CTLA4*
*PDCD1*LG2
*ICAM1*
*CD274*
*STAT1*
*IRF1*
*IFNG*
*GNLY*
*TBX21*
*CCL5*
*LAG3*
*CD247*
*ICOS*
*IL18RAP*
*CXCL9*
*CXCL10*
*HLA-DPB1*
*HLA-DPA1*
*HLA-DMB*
*HLA-DRA*
*HLA-DMA*
*CD80*
*HLA-DOA*
*CD4*
*HAVCR2*

**Figure 1.**
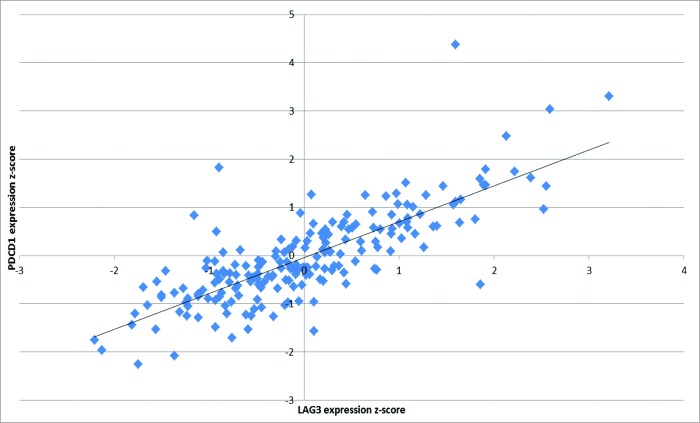
Significant correlation between *LAG3* and *PDCD1* (*PD1*) expression. Pearson correlation analysis indicates the inhibitory molecules *LAG3* and *PDCD1* (*PD1*) are highly coordinated in mRNA expression (R^2^ = 0.623).

Of the 28 genes in the CIRC, 20 have previously been associated with outcome based on experimental data.^1,4^^,^^5^ We show here that these prognostic immune response genes are highly coordinately expressed in CRC and correlate with other biologically associated genes including *HLA-DQA1*, *HLA-DRB5*, *HLA-DPB1*, *LAG3*, TIM3, *CTLA4*, and *CCL5*. The degree of correlation between immune checkpoint receptor/ligand gene expression was notable (**Table S2**). Particularly striking was the correlation between PD1 and LAG3 (r^2^ = 0.62, **Fig. 1**). Given the Th1 bias of the cluster we performed separate unsupervised hierarchical clustering analysis of Th2 cytokines IL2, IL3, IL4, IL5, and IL6 and transcription factor GATA3 (data not shown), revealing that these genes were excluded from the CIRC. We also note that the Th17 cytokine IL17A was also excluded from the cluster (**Fig. S1**).

### Molecular determinants of the co-ordinate immune response cluster

Two-dimensional hierarchical clustering was performed for the entire gene list (**Table S1**) incorporating expression data together with molecular and clinical characteristics (*KRAS*, *BRAF*, *NRAS*, *TP53*, *PIK3CA*, and *PTEN* mutations; microsatellite status, methylation subtype, tumor stage, tumor site, and recurrence data). The microsatellite status of the total patient population was as follows: 11.8% were MSI-H, 15.9% were MSI-L (microsatellite-low), and 71.8% were MSS. 6.7% of patients were *BRAF* mutant *(MT)/MSI-H*, and 3.1% were *BRAF MT/MSS*. The mutational status of the cohort was as follows – *KRAS MT* (39.5%), *NRAS MT* (8.2%), *BRAF MT* (9.7%), *PIK3CA MT* (18.4%), and *TP53 MT* (51.8%). 38.5% of patients were quadruple wild type (*BRAF WT*, *KRAS WT*, *NRAS WT* and *PIK3CA WT*). We also investigated mutations in the novel genes *POLE* (7.2% of patients) and *POLD1* (2.6% of patients), which have recently been highlighted as key drivers of colorectal carcinogenesis for a minority of CRC patients.^16^

**Figure 2.**
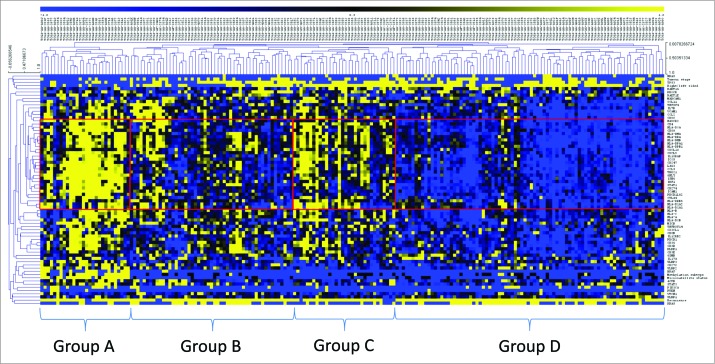
Two-dimensional hierarchical clustering delineates distinct immunological CRC patient groups. Gene expression (yellow, high expression; black, intermediate; blue, low expression) was clustered together with mutation data of key genes (*TP53*, *KRAS*, *BRAF*, *NRAS*, *PI3KCA* and *PTEN* (yellow, mutant; blue, wildtype)) and clinical data (microsatellite status (yellow, MSI-H; black, MSI-L; blue, MSS), recurrence data (yellow, recurred/progressed; blue, disease-free), tumor site (yellow, left sided; blue, right sided), tumor stage (yellow, stage III/IV; blue, stage I/II), methylation subtype (yellow, CIMP-H; black, CIMP-L; blue, CIMP-negative). Clustering was performed by genes/mutations/clinical data (rows) and patients (columns) using the Pearson algorithm. Red boxes indicate groups of patients with strong clustering of the coordinate immune response cluster. Patients were delineated into four distinct groups (**A–D**) on the basis of the dendrogram and the cluster expression.

Hierarchical clustering delineated four distinct patient groups (**Fig. 2**, **Table 2**). Group A patients demonstrated strong CIRC expression (mean expression 0.98). Notably, all of the MSI-H patients were included in this group, who constituted 82.1% of all group A patients, whereas only 10.7% were MSS and 7.1% were MSI-L. Across the entire cohort, expression of the CIRC signature was significantly higher in the MSI-H cancers vs. MSS (*p* < 0.001) and MSI-L cancers (*p *< 0.001). Multivariate analysis revealed that expression of *HLAA*, *HLAB*, and *HLAC* were all significantly less in MSI-H than MSS cancers (*p* = 0.027, *p* = 0.017, and *p* = 0.018, respectively for *HLA-A*, *B* and *C*), consistent with previous observations.^17^ Group A was characterized by the CpG island methylator phenotype (CIMP-H) (67.9%), right-sided tumor site (82.1%), *TP53* wild type (65%) and *KRAS* wild type (82.1%). 50% of patients in this group were *BRAF* mutant and of these *BRAF* mutants, 92.9% were MSI-H. Notably, *POLE* and *POLD1* mutant tumors were associated with higher expression of the CIRC (*p *< 0.05). 42.9% of patients in group A were either *POLE* or *POLD1* MT, and 70.6% of all patients with *POLE* or *POLD1* mutations were found in group A; 100% of *POLD1* mutants were assigned to group A. Of the non-MSI-H patients in group A, 3/5 were *POLE* mutant, two of which were MSS and one MSI-L.

**Table 2.** Characteristics of patient groups. For microsatellite status, methylation and tumor side, the most frequent result is stated. Tumor side refers to the right or left side of the colon. The cluster expression pattern displays the expression pattern of the CIRC cluster in each patient group

	Group A	Group B	Group C	Group D
Microsatellite status	MSI-H (82%)	MSS (86%)	MSS (94%)	MSS (75%) MSI-L (25%)
Methylation	CIMP-High (68%)	CIMP-Neg (77%)	CIMP-Neg (66%)	CIMP-Neg (69%)
Side of tumour	Right (82%)	Left (63%)	Left (94%)	Left (82%)
Stage I+II	73%	55%	72%	49%
TP53 MT	35%	65%	62%	48%
BRAF MT	50%	4%	3%	1%
KRAS MT	18%	47%	22%	49%
NRAS MT	0%	4%	9%	13%
PIK3CA MT	39%	14%	9%	18%
Quadruple WT	18%	39%	69%	33%
Percentage of patients	14%	26%	16%	43%
Mean cluster expression z-score	+0.98	+0.13	+0.50	−0.62
Cluster expression pattern	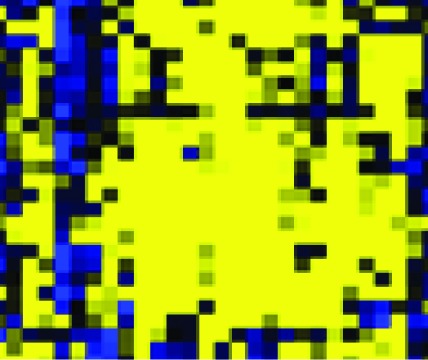	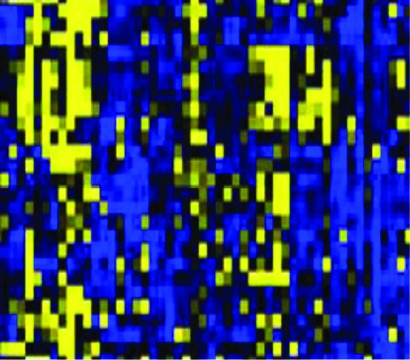	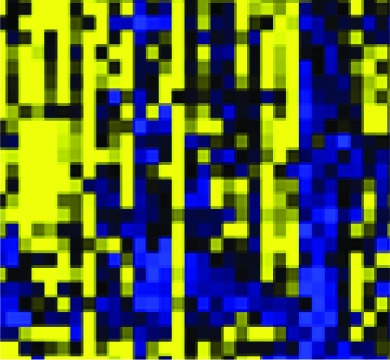	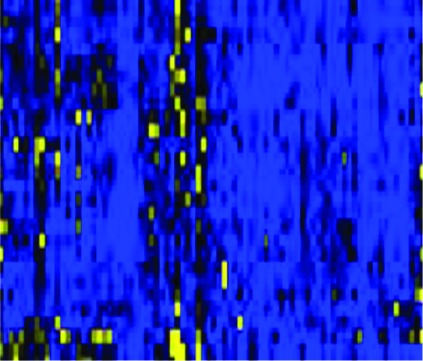

We then analyzed patient groups B, C, and D (**Fig. 2**), which displayed lower expression of the CIRC signature than group A, to determine whether any molecular characteristics were associated with low CIRC expression. Group D comprised cancers with the lowest expression of the cluster (mean expression –0.62) and represented 43% of the entire patient population; group B had a lower mean cluster expression (0.13) than group C (0.50). Significantly, *RAS* mutation (*KRAS*/*NRAS*), occurring in 47.7% of patients, was associated with lower CIRC expression (*p* < 0.001). This was confirmed in the provisional TCGA data set, in which mutation and expression data for a further 30 patients (225 total) is available (*p* < 0.001). The frequency of *RAS* mutation was significantly higher in group B and D than other patient groups (*p* = 0.01), with group D having the highest frequency (D (61.9%)>B (51.0%)>C (28.1%)>A (21.4%)), (*p* = 0.01). Consistent with these observations, within MSS cancers (enriched in groups B–D), there was a strong trend for *RAS* mutation to be associated with low CIRC expression (*p* = 0.076). In group A, 83.3% of *RAS* mutations were MSI-H and 16.7% were MSS (**Table 2**). In *RAS* mutant MSI-H patients, the expression of *HLA-DRA* was lower (0.518) than in *RAS* wild types (1.027) but this difference did not reach significance (*p* = 0.209). Finally, multivariate analysis indicated that *KRAS* and *NRAS* mutation was associated with decreased *CD4* expression (*p* < 0.001) (**Table S3**). The association of low expression of the cluster and *RAS* mutation was particularly strong in the case of *NRAS* mutation where 76.9% of these cancers were in group D and over 90% of these tumors clustered in the two lowest expression groups (B and D). Thus *RAS* mutation, unless linked with microsatellite instability, is commonly associated with low levels of *Th1* infiltration and activation, class II expression and inhibitory checkpoint expression. **Table 3** shows the distribution of key mutations.

**Table 3.** Frequencies of mutations in total patient population and distribution of mutations across patient groups A–D. We classified patients initially on the basis of *KRAS* status, followed by *NRAS*, *BRAF*, and finally *PIK3CA*. According to these criteria, *KRAS* mutations may also have *NRAS*, *BRAF,* and *PIK3CA* mutations (*KRAS* MT +/–*NRAS* MT +/–*BRAF* MT +/–*PIK3CA* MT). *NRAS* mutations are *KRAS* wildtype but may have mutations in *BRAF* and *PIK3CA* (*KRAS* WT + *NRAS* MT +/–*BRAF* MT +/–*PIK3CA* MT). *BRAF* mutants are *KRAS* and *NRAS* wildtype but may have mutations in *PIK3CA* (*KRAS* WT + *NRAS* WT + *BRAF* MT +/–*PIK3CA* MT). *PIK3CA* mutants are wildtype for *KRAS*, *NRAS,* and *BRAF*. Quadruple wildtype patients are *KRAS*, *NRAS*, *BRAF,* and *PIK3CA* wildtype. *TP53* mutation status is independent of other mutations

	% of total patient population	% of total in patient group A	% of total in patient group B	% of total in patient group C	% of total in patient group D
*TP53* MT	51.8%	6.9%	32.7%	19.8%	40.6%
*BRAF* MT	9.2%	72.2%	11.1%	5.5%	11.1%
*KRAS* MT	41.0%	7.5%	30%	10%	52.5%
*NRAS* MT	6.7%	0%	15.4%	7.7%	76.9%
*KRAS*/*NRAS* MT	47.7%	6.5%	28.0%	9.7%	55.9%
*PIK3CA* MT	4.1%	50%	37.5%	0%	12.5%
Quadruple wildtype	39.0%	6.6%	26.3%	28.9%	38.2%

Several groups have identified molecular subtypes of CRC based on expression data. In an effort to create consensus, The colorectal cancer subtyping consortium (CRCSC) have defined an integrated classification – consensus molecular subtypes (CMS).^18^ To investigate how closely our CIRC patient groupings (based predominantly on immunological gene expression) correlate with the recently announced CMS classifications, we retrieved CMS data for the TCGA dataset. **Table 4** shows the distribution of CMS classifications across our four patient groups. As expected, CMS1, which is a group comprised of mostly MSI-H and *BRAF MT* tumors with strong immunity, falls predominantly within Group A. Interestingly, 80% of CMS3 tumors, which are epithelial with *KRAS* mutations, fall within patient groups B and D, similar to our *KRAS* distribution data (**Table 3**). This reinforces the hypothesis that *KRAS* mutant tumors are immunologically bland. A large proportion of CMS4 tumors, which are predominantly mesenchymal with a poor prognosis, were found in patient group B (63.9%), and a large proportion of CMS2 tumors, which are epithelial with high CIN and WNT/MYC pathway activation, fall within patient group D (61.5%). This demonstrates that patient groups B and D are distinct despite both groups having low CIRC expression levels.

**Table 4.** Distribution of Consensus Molecular Subtype (CMS) groups across CIRC patient groups. The percentage of each CMS group that fall within each CIRC patient group. 5.1% of TCGA patients did not have CMS classification data available

	Percentage of total patient population	CIRC Group A	CIRC Group B	CIRC Group C	CIRC Group D
CMS1	13.8%	81.5%	7.4%	3.7%	7.4%
CMS2	40.0%	0.0%	15.4%	23.1%	61.5%
CMS3	10.3%	0.0%	30.0%	20.0%	50.0%
CMS4	18.5%	5.6%	63.9%	13.9%	16.7%
Unclassified	12.3%	12.5%	25.0%	12.5%	50.0%

## Discussion

In this study, we identified a group of tightly co-regulated immune-related genes that we termed the CIRC, and used this to assess differences in the intra-tumoral immune response in a molecularly characterized cohort of CRC patients. The CIRC signature we defined stems from the work of Galon and colleagues, who first established the prognostic impact of T cell infiltration in CRC, initially highlighting seven co-modulated genes principally associated with *Th1*-associated immunity that correlated with outcome.^1^ Subsequently, they demonstrated that the *Th1* genes, *TBX21*, *IFNG*, *IRF1* and *STAT1* were all individually associated with outcome.^4^ Other independent immune gene predictors in that analysis were *IL18RAP*, *ICOS*, PD-L1, PD-L2 and PD-1. We extended this gene list by adding the class I and class II genes, further immune checkpoint genes and a number of other genes related to innate immune recognition and T cell activation, generating an initial gene list of > 50 genes. Unsupervised hierarchical clustering then highlighted a core cluster of 28 tightly co-regulated genes comprising the CIRC signature. Importantly, 20 of the 28 genes had previously been highlighted by Galon and colleagues^1,4^^,^^5^ as either prognostically or predictively relevant, including five of the original prognostic seven gene cluster (with only CD8A (for which microarray data was unavailable) and GZMB not represented). In contrast, genes associated with Th2 and Th17 profiles were excluded from the cluster. Thus, applying a different approach to an independent data-set our analysis verified the high level of correlation of a number of genes based on *Th1*-associated immunity, and also highlighted involvement of additional immune genes.

An important feature of the CIRC signature is that it includes essentially all class II MHC loci, as well as CD4, whereas in contrast, expression of class I MHC molecules, CD8B and also GZMB are all excluded from the signature. In addition to the critical role of T helper cells for CD8^+^ cell priming^19,20^ and expansion,^21, 22^ CD4^+^ cells have also been suggested to be major mediators of immunological tumor cell death.^23-25^ Adoptive CD4^+^ T cell transfer has been found to upregulate class II expression on tumor cells mediating protection from tumor progression.^24^ Class II upregulation was mediated via IFNγ and protection was attenuated using anti-IFNγ antibodies. Recent adoptive transfer approaches involving autologous CD4^+^ T cells have met with clinical success. A durable complete response was obtained in a patient with melanoma after infusion of NY-ESO-1 specific CD4^+^ cells recognizing an HLA-DP4 restricted epitope.^26^ Additionally, a durable response was obtained in a patient with cholangiocarcinoma on infusion of autologous mutation-specific CD4^+^ cells which adopted a poly-functional *Th1* phenotype.^27^ These studies highlight the potential for CD4^+^ cells to mediate clinically potent anti-tumor responses via *Th1* mechanisms. The CD4-centric nature of our CIRC highlights that CD4^+^ T cells may be important in CRC anti-tumor immunity.

The CIRC included the major immune checkpoint molecules. Not only PD-L1, PD-L2 but also LAG3, TIM3 and CTLA4 were all represented in the CIRC and there was a high degree of correlation between inhibitory checkpoint gene expression. This is consistent with the expected feedback sequelae of a pronounced *Th1* infiltrate. IFNγ is the canonical cytokine associated with Th1 T helper cells and expression of PD-L1 is significantly augmented by IFNγ.^28^ *IRF1* is of primary importance in the constitutive expression of PD-L1 and in IFNγ-driven upregulation.^29^ In animal models, significant percentages of infiltrating CD4^+^ and CD8^+^ cells co-express high levels of both PD-1 and *LAG-3*.^30^ Dual anti-LAG3/anti-PD-1 immunotherapy strikingly enhanced survival in the MC38 colon cancer model compared with animals treated with single antibodies alone.^30^ Our data provide justification for trialing combinations of checkpoint blockade agents in CRC.

One of the key aims of this study was to investigate the somatic factors associated with the immune response in CRC. Group A, which exhibited strong expression of the CIRC signature, was dominated by MSI-H tumors, all of which fell in this grouping. This complements very well the work of Biossière-Michot and colleagues, who revealed that MSI-H tumors have a high density of *Tbet*-positive Th1 T cells relative to MSS tumors, and higher expression of the chemokines *CCL5*, *CXCL9*, and *CXCL10*, all of which are found in the CIRC.^12^ The *Th1* response may be driven through activation of the *CXCL9*/*CXCL10* signaling axis.^12^ Previous studies have also shown that MSI-H phenotype is highly associated with class II expression.^31^ Strongly DR positive tumors had a significantly higher TIL density than those with absent or weak staining and survival was significantly better in patients with high DR expression. Consistent with this, Bindea and colleagues demonstrated that the expression of several class II genes, including *HLA-DRA*, are individually associated with improved disease-free survival.^5^ High DR expression in MSI-H CRC contrasts with that of class I molecules, which are completely lost in 60% of sporadic MSI-H cases and only 16.7% of right sided MSS cancers.^17^ Thus, the immune landscape of MSI-H CRC, which is characterized by a high mutational burden including frameshift mutations,^10^ is dominated by T helper cell infiltration and activation, class II expression and coordinated upregulation of a range of immune checkpoint genes. These patients may therefore have greater responses to checkpoint blockade therapies. Similarly, *POLE*/*POLD1* mutant tumors, which also have a high mutational burden,^16^ were also associated with high CIRC expression. These data support the hypothesis that high mutational burden may translate into a strong immune response, possibly due to neoantigen presentation, leading to better outcomes. The finding that the gene encoding TGFβR undergoes frameshift mutation in 90% of MSI-H CRC, giving rise to a highly immunogenic promiscuous class II peptide, is entirely in keeping with this hypothesis.^32^

*RAS* mutation was significantly associated with lower CIRC expression, with over 60% of cancers in the group exhibiting very low expression (group D) being *RAS* mutant. *KRAS* and *NRAS* mutant CRC had significantly lower levels of CD4^+^ T cells on multivariate analysis. Although 21.4% of cancers in group A were *RAS* mutant, over 80% of these were MSI-H. This is the first analysis to our knowledge to clearly define the immunological landscape of *RAS* mutant CRC. Ogino and colleagues examined the interaction between T cell infiltration and *KRAS*, *BRAF,* and *PIK3CA* mutation status and found no significant associations with *KRAS* mutation,^33^ but this study was limited to the density of CD3^+^, CD8^+^, CD45RO^+^, and *FoxP3*^+^ cells. Morris and colleagues demonstrated that transfection of mutant *KRAS* into fibroblasts abrogated the IFNγ -mediated upregulation of class II expression: effects on class I expression were minimal.^34^ *RAS* transfection inhibited proliferation and IFNγ production of alloreactive T cells, an effect mediated by loss of class II expression on target cells.**^35^** These data suggest a possible mechanism for our observed paucity of class II expression and hence *Th1*-related gene expression in *RAS* mutant CRC. Our data complement previous studies demonstrating widespread abnormalities of class I presentation in *RAS* mutant CRC.^36^ In MSI-H tumors, it is likely that any inhibitory effect of *RAS* mutation is overcome by the strong immunity resulting from neoantigens linked to mutations induced by microsatellite instability. Thus MSI-H *RAS* mutant tumors retain strong *Th1* immunity, despite having numerically lower *HLA-DRA* expression than MSI-H *RAS* wildtype tumors. In sum, these data suggest that the micro-environment of *RAS* mutant CRC is relatively immunologically unfavorable to conventional αβ T cell responses.

An important consideration is how the CIRC signature relates to immunosuppressive pathways. Though *FOXP3* was not in the hierarchical clustering due to an absence of microarray data, we analyzed RNA sequencing data which showed that *FOXP3* expression is highest in patient group A and lowest in group D (data not shown). Additionally, the highly immunosuppressive cytokine IL10 also exhibited high expression in this patient group (data not shown), both consistent with increased inhibitory immunoregulation following establishment of a *Th1*-polarized microenvironment. We also analyzed expression of *STAT3*, which has been linked to enhanced levels of tumor-associated macrophages, myeloid-derived suppressor cells, and poor T cell responses.^37,38^ Although not strongly linked to the CIRC, we found increased *STAT3* expression was associated with *PIK3CA* mutation (**Table S3**). Conditioned media from *PIK3CA* mutant cells has been found to drive *STAT3* activation^39^; Aspirin decreases *STAT3* activation in murine CRC models and promotes apoptosis. Our findings may therefore partly explain previous molecular epidemiology studies^40-42^, which have demonstrated that aspirin usage reduces the risk of CRC recurrence following resection in patients with CRC harboring *PIK3CA* mutations.

The CIRC molecular associations relate to the CRCSC CMS and their immune and molecular characteristics.^18^ The majority of CMS1 patients (MSI high and immune activated) fall within CIRC group A. 82.5% 0f *KRAS* mutated patients were in CIRC groups D and B (the lowest two CIRC expression groups) and almost the same percentage of CMS3 patients, characterized by *KRAS* mutations and no significant immune infiltration and activation, were present in CIRC groups D and B.

In conclusion, we have shown that MSI-H and POL mutant CRC is associated with high-level expression of a coordinated immune response cluster characterized by T helper cell and class II related genes together alongside a range of chemokines and immune inhibitory checkpoint molecules. In contrast, *RAS* mutant tumors have significantly lower expression of this cluster. These findings have potential therapeutic implications. MSI-H tumors may be particularly amenable to CD4^+^ cell expansion and adoptive transfer approaches. Second, although there is interest in the use of immune checkpoint inhibitors in CRC, the coordinated upregulation of immune checkpoint genes suggests that a combinatorial approach is likely to be more successful. Finally, any immunologically based therapy in MSS CRC aimed at *RAS* mutant patients must take into account the relatively immunologically quiescent status of the micro-environment in the majority of *RAS* mutant CRC.

## Methods

### Gene identification

Genes associated with innate and adaptive tumor immunity in cancer^1,4^ and genes relevant to established colon cancer pathways were shortlisted as initial genes. This list was expanded using molecular network pathway analysis.^43-45^ This revealed further molecules and genes with known genetic, pathway and functional associations with our initial genes. Genes revealed through this approach were screened for inclusion into our final gene list based on known immunological relevance (**Table S1**).

### Data extraction

Normalized Agilent microarray and RNAseq z-score data for initial genes and mutation data for *TP53*, *KRAS*, *BRAF*, *NRAS*, *HRAS*, *PIK3CA* and *PTEN* were extracted from TCGA colorectal data set^46^ using the cBioportal tool.^43-45^ Where expression data were unavailable, genes were excluded from the analysis. Clinical data for these patients were retrieved from the TCGA portal (https://tcga-data.nci.nih.gov/tcga/) and tabulated with genetic data. CMS data were obtained from the CRCSC through the Synapse platform (https://www.synapse.org/, Sage Bionetworks).

### Data analysis

Data were tabulated in Excel (Microsoft Corp.) and unsupervised two-dimensional hierarchical clustering was performed using MeV (Dana-Farber Cancer Institute, Boston, MA, USA) and the Pearson correlation. Patient and gene clusters were identified by varying gene-tree distance-thresholds and visual analysis. Normality of distributions was confirmed with the Anderson–Darling test. Pearson Coefficient of determination (R^2^) values were calculated in Excel and Minitab (Minitab Inc.) to investigate correlations in gene expression. Gene expression was correlated against β actin as a control gene. T tests were performed for univariate analyses of normally distributed data, and Mann–Whitney U tests for non-normally distributed data. Multivariate linear regression analyses of gene expression against mutations in key tumor associated genes (P53, *KRAS*, *BRAF*, *NRAS*, *HRAS*, *PI3KCA*, *PTEN*) were completed in Stata 12.2 (Statacorp.). For two by two comparisons, a Chi-squared test was used; if any groups contained less than five values, the Fisher's Exact test was used in preference.

## Supplementary Material

976052_Supplementary_Materials.zip
