# Supplementary material for: An immunogenomic stratification of colorectal cancer: Implications for development of targeted immunotherapy
Source: Oncoimmunology. 2015 Apr 2;4(3):e976052. doi: 10.4161/2162402X.2014.976052 (PMC4404815; doi:10.4161/2162402X.2014.976052)
Supplement: 976052_Supplementary_Materials.zip [file koni-04-e976052-s001.zip › 976052_Tables S1-S3.docx]

**Legends**

Supplementary figure 1: Two-dimensional hierarchical clustering reveals a closely co-ordinated immunological gene expression cluster – The Co-ordinate Immune Response Cluster (CIRC). Clustering was performed by gene expression (rows) and patients (columns) using the Pearson algorithm. Yellow represents high gene expression, black represents intermediate gene expression and blue represents low gene expression. The red box shows a group of closely associated genes (the CIRC) that had a co-ordinated expression pattern across the patient population.

Supplementary table 1: Gene list for analysis

Supplementary table 2: R^2^ values correlating expression of inhibitory molecules in colorectal cancer. Analysis is controlled against beta actin.

Supplementary table 3: Multivariate analysis of gene expression changes in key mutation groups. P values are derived from multivariate linear regression analysis.

**Supplementary figure 1: Separate file**

**Supplementary table 1:**

| **Gene IDs** | |
| --- | --- |
| ACTB | HLA-DQA2 |
| CCL11 | HLA-DRA |
| CCL2 | HLA-DRB5 |
| CCL5 | ICAM1 |
| CD247 | ICOS |
| CD274 | IFNG |
| CD276 | IL12RB2 |
| CD3D | IL17A |
| CD3E | IL18RAP |
| CD3G | IL7R |
| CD4 | IRF1 |
| CD80 | KLRK1 |
| CD86 | LAG3 |
| CD8B | MADCAM1 |
| CTLA4 | MICB |
| CX3CL1 | PDCD1 |
| CXCL10 | PDCD1LG2 |
| CXCL9 | PROCR |
| GNLY | RAET1E |
| GZMB | RAET1G |
| HAVCR2 | STAT1 |
| HLA-A | STAT3 |
| HLA-B | TBX21 |
| HLA-C | TNFRSF14 |
| HLA-DMA | TNFSF4 |
| HLA-DMB | ULBP1 |
| HLA-DOA | ULBP2 |
| HLA-DOB | ULBP3 |
| HLA-DPA1 | VCAM1 |
| HLA-DPB1 | VTCN1 |
| HLA-DQA1 |  |

**Supplementary table 2:**

|  |  | **CTLA4** | **PD1** | **PDL1** | **PDL2** | **TIM3** | **LAG3** | **Beta Actin** |
| --- | --- | --- | --- | --- | --- | --- | --- | --- |
|  |  | **CTLA4** | **PDCD1** | **CD274** | **PDCD1LG2** | **HAVCR2** | **LAG3** | **ACTB** |
| **CTLA4** | **CTLA4** | X | 0.36 | 0.42 | 0.35 | 0.35 | 0.42 | 0.04 |
| **PD1** | **PDCD1** | 0.36 | X | 0.45 | 0.31 | 0.45 | 0.62 | 0.04 |
| **PDL1** | **CD274** | 0.42 | 0.45 | X | 0.48 | 0.5 | 0.51 | 0.07 |
| **PDL2** | **PDCD1LG2** | 0.35 | 0.31 | 0.48 | X | 0.49 | 0.31 | 0.08 |
| **TIM3** | **HAVCR2** | 0.35 | 0.45 | 0.5 | 0.49 | X | 0.52 | 0.06 |
| **LAG3** | **LAG3** | 0.42 | 0.62 | 0.51 | 0.31 | 0.52 | X | 0.03 |
| **Beta Actin** | **ACTB** | 0.04 | 0.04 | 0.07 | 0.08 | 0.06 | 0.03 | X |

**Supplementary table 3:**

| Mutation | Gene Expression | Direction of change | P-value |
| --- | --- | --- | --- |
| BRAF | CD247 | Down | <0.001 |
|  | CD80 | Up | 0.017 |
|  | GNLY | Up | 0.031 |
|  | HAVCR2 | Up | 0.039 |
|  | HLAA | Up | 0.004 |
|  | HLAB | Down | 0.003 |
|  | HLADOA | Down | 0.017 |
|  | LAG3 | Up | <0.001 |
| KRAS | CD4 | Down | 0.008 |
| NRAS | CCL5 | Up | 0.002 |
|  | CD247 | Down | 0.007 |
|  | CD4 | Down | 0.036 |
|  | CXCL10 | Down | 0.037 |
| PIK3CA | CD4 | Down | 0.001 |
|  | HAVCR2 | Up | 0.005 |
|  | STAT3 | Up | 0.002 |
| TP53 | CD274 | Up | 0.028 |
|  | CD276 | Down | 0.001 |
|  | CD4 | Up | 0.005 |
|  | CX3C11 | Down | 0.012 |
|  | CXCL10 | Up | 0.008 |
|  | HLAB | Up | 0.024 |
|  | HLADQ2 | Down | 0.002 |
|  | IFNG | Down | 0.004 |
|  | IL7R | Down | 0.018 |
| PTEN | CD4 | Down | 0.003 |
|  | CTLA4 | Up | <0.001 |
|  | HLADMB | Up | <0.001 |
|  | HLADRA | Down | 0.027 |
|  | IL7R | Down | 0.004 |
|  | STAT1 | Down | 0.001 |
|  | STAT3 | Up | 0.026 |
|  | VTCN1 | Up | 0.017 |
