# Supplementary figures and images for: An immunogenomic stratification of colorectal cancer: Implications for development of targeted immunotherapy
Source: Oncoimmunology. 2015 Apr 2;4(3):e976052. doi: 10.4161/2162402X.2014.976052 (PMC4404815; doi:10.4161/2162402X.2014.976052)

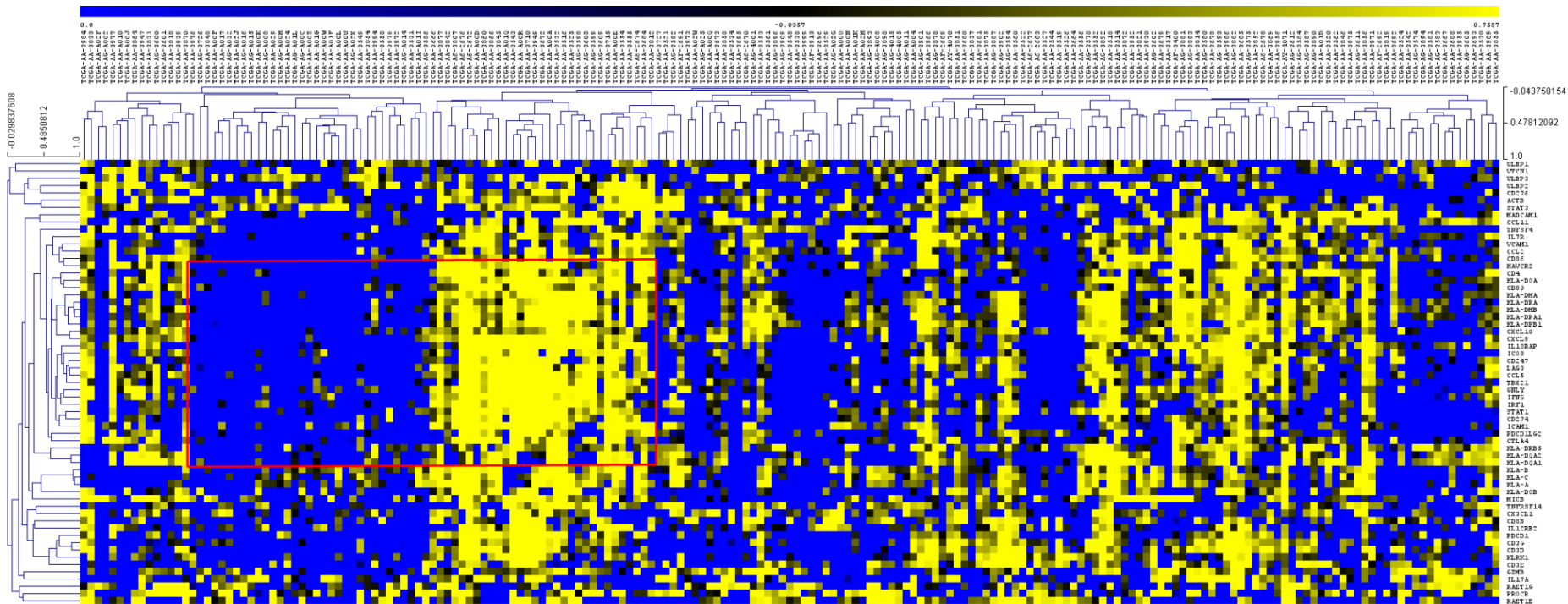

Supplement: 976052_Supplementary_Materials.zip [file koni-04-e976052-s001.zip › 976052_Figure S1.pdf]
